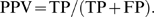# Analyses and Comparison of Imputation-Based Association Methods

**DOI:** 10.1371/journal.pone.0010827

**Published:** 2010-05-26

**Authors:** Yu-Fang Pei, Lei Zhang, Jian Li, Hong-Wen Deng

**Affiliations:** 1 Key Laboratory of Biomedical Information Engineering, Ministry of Education and Institute of Molecular Genetics, School of Life Science and Technology, Xi'an Jiaotong University, Xi'an, People's Republic of China; 2 School of Medicine, University of Missouri-Kansas City, Kansas City, Missouri, United States of America; 3 Center of System Biomedical Sciences, Shanghai University of Science and Technology, Shanghai, People's Republic of China; Peninsula Medical School, United Kingdom

## Abstract

Genotype imputation methods have become increasingly popular for recovering untyped genotype data. An important application with imputed genotypes is to test genetic association for diseases. Imputation-based association test can provide additional insight beyond what is provided by testing on typed tagging SNPs only. A variety of effective imputation-based association tests have been proposed. However, their performances are affected by a variety of genetic factors, which have not been well studied. In this study, using both simulated and real data sets, we investigated the effects of LD, MAF of untyped causal SNP and imputation accuracy rate on the performances of seven popular imputation-based association methods, including MACH2qtl/dat, SNPTEST, ProbABEL, Beagle, Plink, BIMBAM and SNPMStat. We also aimed to provide a comprehensive comparison among methods. Results show that: 1). imputation-based association tests can boost signals and improve power under medium and high LD levels, with the power improvement increasing with strengthening LD level; 2) the power increases with higher MAF of untyped causal SNPs under medium to high LD level; 3). under low LD level, a high imputation accuracy rate cannot guarantee an improvement of power; 4). among methods, MACH2qtl/dat, ProbABEL and SNPTEST perform similarly and they consistently outperform other methods. Our results are helpful in guiding the choice of imputation-based association test in practical application.

## Introduction

The advance in high-throughput genotyping technologies has promoted large-scale genetic association studies aiming to identify genetic variants predisposing to complex diseases [1], [2]. The successful utility of genome-wide association studies (GWAS) has identified a large quantity of SNPs predisposing to a variety of complex diseases, e.g., type 2 diabetes, obesity, and osteoporosis [3], [4], [5]. However, the throughput of most commercial genotyping platforms remains relatively unsatisfactory compared to the total available SNPs across the human genome. As an example, the Affymetrix SNP 6.0 assay contains approximately 1,000K SNPs, which account only for one third of the total number of over three million SNPs identified by the HapMap project [6], [7]. Disease-causing variants may exist within those untyped SNPs which, when typed, could be more informative than their flanking typed SNPs [8].

A potential solution for the problem of low genotyping coverage is to impute untyped SNPs from their nearby genotyped SNPs through their linkage disequilibrium (LD) pattern. It has been shown that association analyses with imputed genotypes can boost the signal over that obtained by analyses of typed genotypes only. Imputed genotype-based association can also be more powerful than tagging approaches which test only single SNPs or small haplotypes of SNPs in a genotyping chip [9], [10], [11], [12].

Several effective genotype imputation methods have been proposed [9], [17], [18]. These methods can produce a probability vector for the three possible genotypes. A critical issue with imputed genotypes is how to integrate them effectively into association analyses. One can use these posterior probabilities directly or pick up the “best-guess” genotype to perform the subsequent association analysis. Several specialized methods have been proposed to model imputed genotypes into association framework [9], [13], [19], [20], [21]. Nonetheless, little is known about their relative performances, and investigators may wonder which methods should be adopted in a particular application. A variety of factors have influences on imputation accuracy [22], but not necessarily on subsequent association tests. A comprehensive comparison among methods must take these influential factors into account. Among previous studies, Marchini and Howie [23] demonstrated the improved power of their method IMPUTE/SNPTEST by comparing it with SNPMStat. However, their comparison was evaluated on a relatively small number of selected data sets under relatively limited conditions. Guan & Stephens [24] studied the effect of imputation accuracy on association power, however their study was performed solely on the software BIMBAM. Hao et al. [25] compared the performance of MACH and Beagle, but not included other popular methods.

Additional comparisons were also conducted when new methods were proposed [13], [19]. Nonetheless, none of them was conducted in a systematic and comprehensive manner. We thus perceive a substantial need to evaluate and compare the performances of most popular imputation-based association methods in a variety of conditions, in order to provide guidance for real applications.

In this study, using both simulated and real data sets, we evaluated the effects of several influential factors on the performances of several imputation-based association methods. These factors include LD level, minor allele frequency (MAF) of untyped causal SNP, and imputation accuracy rate (AR). We selected seven popular methods for investigation, including MACH2qtl/dat, SNPTEST, ProbABEL, Beagle, Plink, BIMBAM and SNPMStat. We also compared their performance under various conditions.

## Results

### Type-I error rates

Type-I error rates are listed in Table 1 and Table 2. When testing association at the imputed potential causal SNP, all methods had correct type-I error rates that were close to the target level 5% under all conditions. When testing for the entire region, all but SNPMStat remain to have reasonable error rates, whereas SNPMStat had an inflated type-I error rate under low LD level. However, when testing for the entire region under high LD level, all methods were conservative. We thus estimated testing accuracy and positive prediction value (PPV) for each method (please refer to the method section for the definitions of accuracy and PPV) as well when testing for the entire region, in order to make methods comparable.

**Table 1:** Type-I error rates of various imputation-based association methods for the causal SNP under the significant level of 5%.

	Quantitative Trait			Qualitative Trait		
	Low-LD	Medium-LD	High-LD	Low-LD	Medium-LD	High-LD
Genotyped-1 SNP/6kb	5.0	5.2	5.1	4.9	5.1	4.3
Ideal	5.1	5.0	5.0	5.0	5.1	5.1
SNPTEST	5.0	5.0	4.8	5.0	5.0	4.8
SNPTEST-BG	5.0	5.0	5.1	5.0	5.1	5.1
MACH2qtl/dat	5.0	5.0	5.0	4.9	4.9	5.0
BIMBAM	5.0	4.8	5.0	5.0	4.8	5.0
Beagle	-	-	-	4.9	5.1	4.9
Plink	-	-	-	4.4	4.3	4.4
ProbABEL	5.1	5.1	5.0	5.1	5.2	5.1
SNPMStat	-	-	-	7.0	6.0	4.7

**Table 2:** Type-I error rates of various imputation-based association methods for the test region under the significant level of 5%.

	Quantitative Trait			Qualitative Trait		
	Low-LD	Medium-LD	High-LD	Low-LD	Medium-LD	High-LD
Genotyped-1 SNP/6kb	5.0	5.1	4.7	5.5	5.3	3.8
Ideal	5.1	5.1	4.6	4.9	5.1	2.2
SNPTEST	5.7	5.6	3.6	4.8	3.9	2.6
SNPTEST-BG	4.8	5.1	3.8	4.2	5.1	2.8
MACH2qtl/2dat	4.4	4.3	2.9	4.9	2.7	1.8
Beagle	-	-	-	4.5	3.5	1.8
Plink	-	-	-	4.4	5.2	2.0
ProbABEL	3.8	5.1	3.2	5.0	5.1	2.2
SNPMStat	-	-	-	8.4	7.3	1.6

### Power estimates

Table 3 and Table 4 list power estimate, accuracy and PPV when testing for the entire region. For both quantitative and qualitative traits, MACH2qtl/dat, ProbABEL and SNPTEST had the best performance under most situations, followed by SNPTEST-BG. Beagle had similar performance to SNPTEST-BG under high LD level, but was inferior under medium LD level. SNPMStat and Plink had the lowest power. As BIMBAM estimated *p*-value through permutation with 1,000 replicates, its output had a resolution 1.0e-03, which did not reach the significant level (2.0e-04) with Bonferroni correction. We thus did not include BIMBAM in the analysis for the entire region.

**Table 3:** Power estimates of various imputation-based association methods for testing the whole region under the significant level of 5%.

		Quantitative Trait			Qualitative Trait		
		Low-LD	Medium-LD	High-LD	Low-LD	Medium-LD	High-LD
Power	Genotyped-1 SNP/6kb	5.3	10.7	29.6	5.1	8.2	23.7
	Ideal	32.8	34.8	39.0	22.3	24.5	28.1
	SNPTEST-BG	4.8	10.6	35.6	4.9	9.1	27.2
	SNPTEST	5.5	14.0	38.1	4.9	10.3	27.3
	MACH2qtl/2dat	5.1	12.3	35.5	4.8	10.1	27.2
	Beagle	-	-	-	5.0	6.1	25.5
	Plink	-	-	-	4.9	7.6	15.8
	ProbABEL	5.2	12.8	36.3	5.2	10.5	27.9
	SNPMStat	-	-	-	7.2	9.2	20.6

**Table 4:** Accuracy and positive prediction value (PPV) estimates of various imputation-based association methods for testing the whole region under the significant level of 5%.

		Quantitative Trait			Qualitative Trait		
		Low-LD	Medium-LD	High-LD	Low-LD	Medium-LD	High-LD
Accuracy	Genotyped-1 SNP/6kb	50.1	52.8	62.4	49.8	51.5	60.0
	Ideal	63.8	64.9	67.3	58.7	59.7	62.9
	SNPTEST-BG	49.6	52.8	65.5	50.1	52.3	62.2
	SNPTEST	49.9	54.2	67.2	50.1	53.2	62.4
	MACH2qtl/2dat	50.4	54.0	66.3	50.0	53.7	62.7
	Beagle	-	-	-	50.3	51.3	61.9
	Plink	-	-	-	50.3	51.2	56.9
	ProbABEL	50.7	53.9	66.6	50.1	52.7	62.9
	SNPMStat	-	-	-	50.5	51.2	59.0
PPV	Genotyped-1 SNP/6kb	51.3	67.8	86.2	47.9	60.8	86.2
	Ideal	86.6	87.3	90.1	82.0	82.8	92.7
	SNPTEST-BG	45.8	67.5	88.4	51.1	67.3	90.7
	SNPTEST	49.1	71.4	91.3	50.7	72.5	91.3
	MACH2qtl/2dat	53.7	74.1	92.4	49.5	78.9	93.8
	Beagle	-	-	-	52.6	63.5	93.4
	Plink	-	-	-	52.7	59.4	88.8
	ProbABEL	57.8	71.5	91.9	51.0	67.3	92.7
	SNPMStat	-	-	-	51.5	56.1	84.6

**Figure 1:**
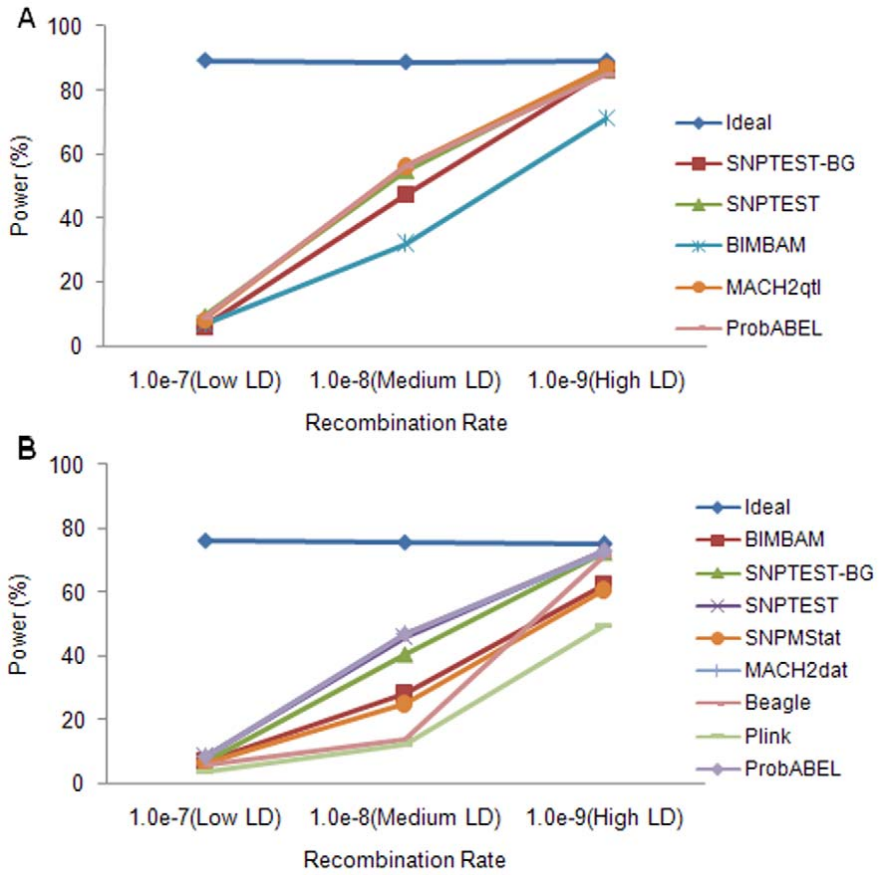
The effect of LD level on power estimate. (a) Quantitative traits; (b) Qualitative traits.

Figure 1 displays power estimate when testing at a single SNP. For both quantitative and qualitative traits, power increased with increasing LD level. For example, the power of SNPTEST when analyzing quantitative trait was 9.2% at low LD level, then increased to 54.7% at medium LD level, and reached 86.5% at high LD level. Among methods, MACH2qtl/dat, ProbABEL and SNPTEST performed similarly and in general produced the highest power, followed by SNPTEST-BG, BIMBAM and SNPMStat. Beagle had similar performance to SNPTEST-BG under high LD level. But under medium LD level, it has similar performance to Plink and was not as good as other methods. Plink had a lower power than other methods under all LD levels.

**Figure 2:**
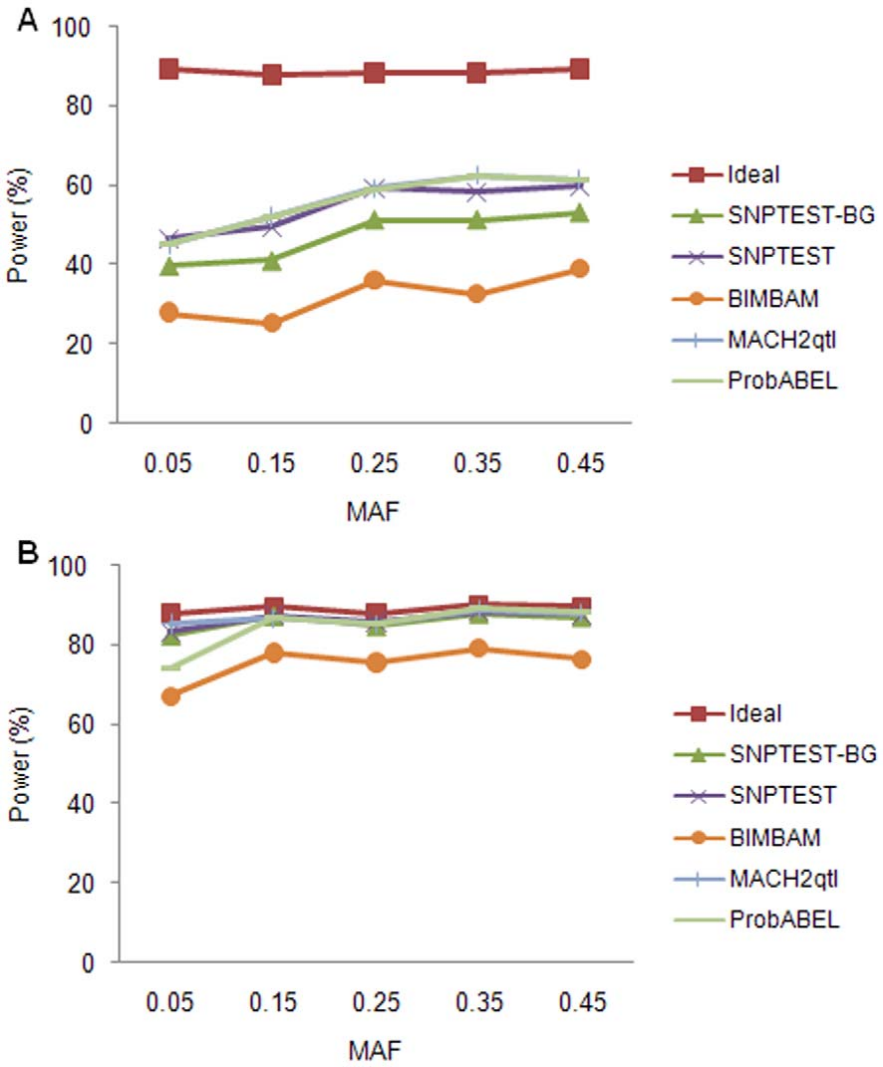
The effect of MAF on power estimate for quantitative trait. (a) Medium LD level; (b) High LD level.

Figure 2 displays the influence of MAF of untyped causal SNP on power estimates when analyzing quantitative traits at a single SNP. Under low LD level, only a small portion of imputed SNPs could pass the quality control. Consequently, the number of SNPs passing the QC under each MAF interval was close to zero. Therefore, we report only the results under medium and high LD levels. Under medium LD level, power increased with increasing MAF interval. For example, when the MAF interval increased from 0.05 to 0.45, the power of SNPTEST increased from 46.6% to 59.8%. Among methods, MACH2qtl, ProbABEL and SNPTEST again had the highest power, followed by SNPTEST-BG and BIMBAM. Under high LD level, all methods but BIMBAM had similar power which maintained at high rates ranging from 81.8% to 88.4%, while that of BIMBAM ranged from 67.3% to 79.2%.

**Figure 3:**
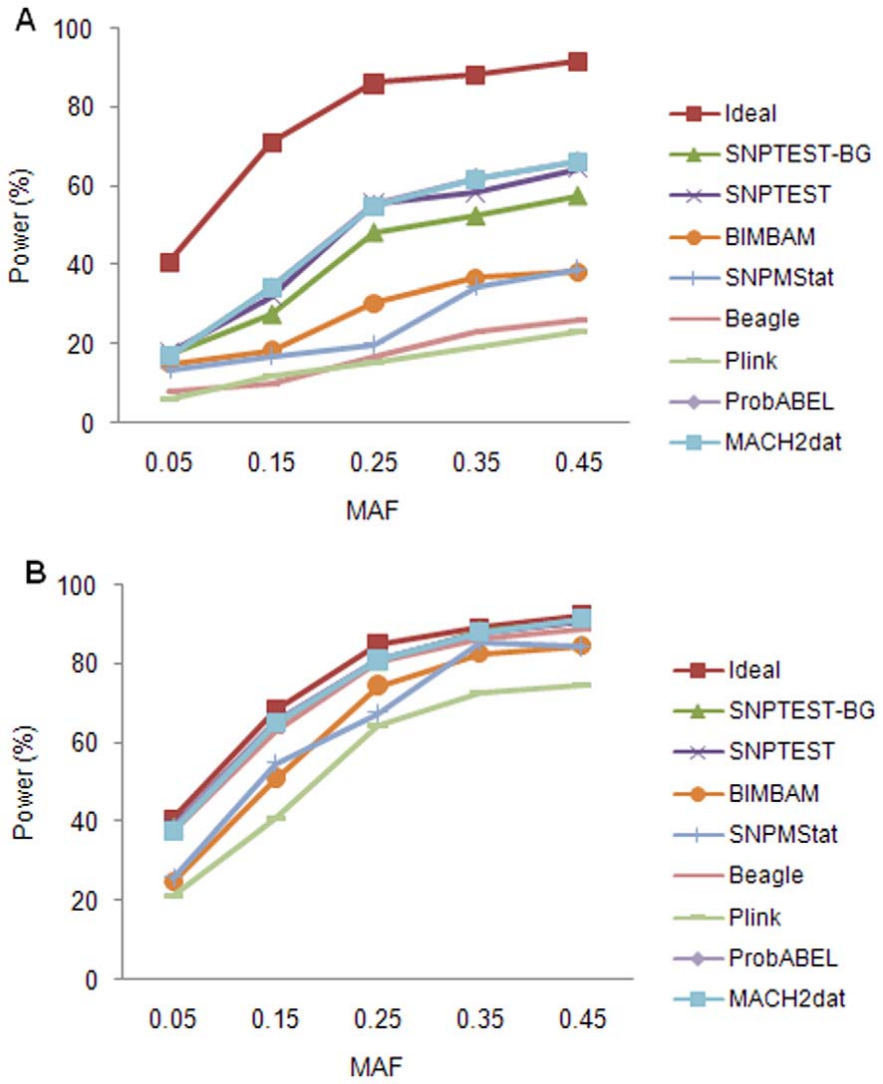
The effect of MAF on power estimate for qualitative trait. (a) Medium LD level; (b) High LD level.

The influence of MAF when analyzing qualitative trait at a single SNP is shown in Figure 3. The trends in power estimates were similar to that for quantitative trait. In this case, power of all methods under high LD level clearly increased with increasing MAF interval. Among methods, MACH2dat, ProbABEL and SNPTEST again had the highest power in most situations, followed by SNPTEST-BG. Beagle had similar performance to SNPTEST-BG under high LD level, but was inferior under medium LD level. SNPMStat and BIMBAM had approximately equal powers, which were higher than that of Beagle under medium LD level, but lower under high LD level. Plink was not as good as other methods under all situations.

**Figure 4:**
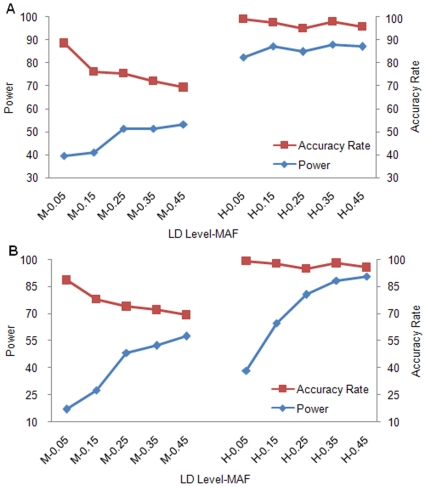
The effect of imputation accuracy rate on power estimate. Each label along x-axis represents a specific combination of LD level and MAF. Within each label, the first letter “M” and “H” refer to, respectively, medium and high LD level. (a) Quantitative trait; (b) Qualitative trait.

We then evaluate the influence of imputation accuracy rate (AR) on power estimate. AR was defined as the number of correctly imputed genotypes divided by the total number of untyped genotypes. As an illustration, we selected SNPTEST to analyze because it performed well in terms of both AR and power. Figure 4a displays the power estimate for quantitative trait. It was clear that under medium LD level, power increased from 39.6% to 53.2% with AR decreasing from 88.6% to 69.3%. Under high LD level, both power and AR maintained at high levels. The trends for qualitative trait were similar, as shown in Figure 4b.

### Real data

**Figure 5:**
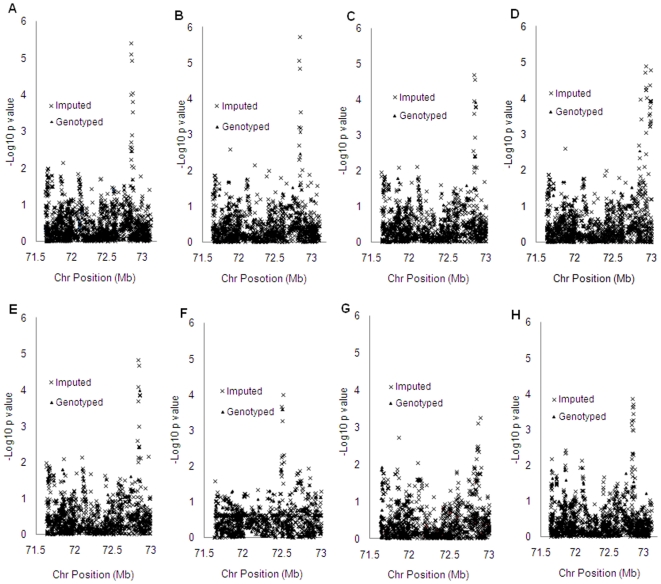
Application to real data set. (a) SNPTEST; (b) SNPTEST-BG; (c) MACH2dat; (d) SNPMStat; (e) ProbABEL; (f) Beagle; (g) Plink; (h) BIMBAM.

Figure 5 displays the results when analyzing the real data set described in the method section. All methods boosted the signal at imputed markers. The minimum *p*-values for MACH2dat, SNPTEST-BG, SNPTEST, ProbABEL and SNPMStat were close to 1.0e-05 and those for Beagle, BIMBAM (based on 100,000 permutations) and Plink were close to 1.0e-04.

### Running Time

Running time was measured on a Linux cluster with 4 computation nodes, each having two Intel Xeon Quad-core processors and 7 GB RAM. Time for performing association tests in a 250kb region with 1,000 subjects was recorded and converted to that with a single core processor. For ProbABEL, running time was measured as the sum of imputation time by MACH and association test computation time. All the methods completed analysis within 15 minutes. Running time for MACH2qtl/dat, SNPTEST-BG, SNPTEST, ProbABEL, Beagle, Plink, BIMBAM and SNPMStat was 13.2, 13.1, 13.2, 13.2, 2.1, 1.0, 4.0 and 4.2 minutes, respectively.

## Discussion

In this study, using both simulated and real data sets, we investigated and compared the performances of seven imputation-based association methods: MACH2qtl/dat, SNPTEST, ProbABEL, Beagle, Plink, BIMBAM and SNPMStat under a variety of conditions. Our conclusions include: 1). all the investigated methods can boost signals with imputed genotypes, and the power of association improves under medium and high levels of LD, with the magnitude of power increase depending on the strength of LD; 2) the power increases with increasing MAF of untyped causal SNPs under medium LD level; 3). high imputation AR cannot guarantee a power improvement in regions with low LD level; 4). among methods, MACH2qtl/dat, SNPTEST and ProbABEL have similar performance and have higher power than other methods for both quantitative and qualitative traits.

On testing association in regions with low or medium LD level, SNPMStat has an inflated type-I error rate. This phenomenon is likely to be caused by the fact that SNPMStat uses only a small number of SNPs to impute, which may lead to a less accurate imputation in low or medium LD level and, consequently, to a higher genotyping error that could inflate type-I error of association test [26]. We also observed that all methods had type-I error rates that were lower than the target level when testing association for the entire region under high LD level, which is probably caused by the overly conservative Bonferroni correction when applied to highly correlated tests.

Statistical power of association test is influenced by a variety of attributes of data, e.g., locus effect, MAF, LD level, and sample size [27]. Obviously, testing markers has inferior power than testing the causal SNP itself [8], [9]. However, causal SNPs are usually not genotyped. Alternatively, genotype imputation takes chance to observe genotypes on more representative markers or causal SNPs themselves, and thus sheds light on power improvement over tests on genotyped SNPs only. Nonetheless, the pattern in power improvement is context dependent and complicated. In our simulation, power increased with increasing LD level and/or MAF of untyped causal SNP in regions with a medium LD level. In order to interpret this pattern, we calculated the MAF of imputed and causal SNPs, and the LD measure D′ between them. Under low, medium and high LD levels, the average values of D′ were 0.21, 0.66 and 0.95, respectively, and the average MAF discrepancies were 0.07, 0.03 and 0.006, respectively. Low LD level virtually eliminates any detectable signal [27] and results in a power that was close to type-I error rate. Medium LD level, on the other hand, could retain some association signal through imperfect LD, resulting in a power that was lower than that on causal SNPs. Additionally, the power is influenced by MAF in that the discrepancy of allele frequency between imputed and causal SNPs becomes narrower as MAF increases, resulting in an increase of power [27]. High LD level assures nearly perfect match between imputed and causal SNPs, and thus retains substantial portion of the true effect size, resulting in power reaching nearly to that by testing on causal SNPs under all MAF intervals.

Our results show that in regions with medium or high LD level, the imputation-based test can improve the association power even under moderate imputation AR (69.2% under medium LD level). This conclusion agrees with that of Guan and Stephens, who indicated that imputation-based methods could be robust to imputation AR and could improve power to detect associations even when average imputation accuracy was not perfect. However, our results also show that high imputation accuracy cannot guarantee an improvement of power in regions with low LD level. Our previous study has shown that imputation accuracy is primarily determined by the LD between imputed and typed SNPs, and their MAF. Under low LD level, though the LD level across the entire region is low, SNPs with small MAF may exhibit locally high LD level, resulting in high imputation accuracy. However, association tests are usually low-powered in regions with low LD level, as demonstrated by our simulations. This discordance between our study and that of Guan and Stephens is partially due to the fact that the real data sets they used may exhibit on average higher LD levels than the simulated data sets in our study.

Different methods use imputed genotypes differently for association tests. SNPTEST and SNPTEST-BG use the imputed genotypes from the software IMPUTE as input. SNPTESTBG utilizes the “best-guess” genotype, while SNPTEST considers the uncertainty and takes the posterior probability into analyses. The superior of SNPTEST to SNPTEST-BG demonstrates that incorporating the uncertainty of imputation can improve association power. BIMBAM tests association on the mean-genotype from the software fastPHASE. fastPHASE was not as good as IMPUTE in terms of AR [22]. When the LD level is fixed, low AR weakens the correlation between imputed and original SNPs and enlarges the discrepancy between allele frequencies. For example, under medium LD level, the average D′ between imputed and original SNPs was 0.57 for fastPHASE (0.66 for IMPUTE) and the average allele frequency discrepancy for fastPHASE was 0.06 (0.03 for IMPUTE). Therefore, the power of BIMBAM was not as good as SNPTEST-BG. Beagle performed similarly to IMPUTE under high LD level, but was inferior under medium LD level in terms of AR. Therefore, the power of Beagle was similar to that of SNPTEST-BG under high LD level, but inferior under medium LD level.

Among methods, all but SNPMStat implement a two-stage approach: 1) imputing untyped genotypes; 2). performing association tests on imputed genotypes. In these methods, all individuals are assumed to be randomly sampled from a “population”. However, affected subjects are more likely to be more closely related to each other than this assumption would imply [13]. To circumvent this assumption, SNPMStat implements a maximum likelihood (ML) approach to integrate genotype imputation and association simultaneously. This approach, by taking disease status into account when imputing, could theoretically lead to more accurate imputation and powerful association test. However, the computation of fitting a joint model of genotype and phenotype is challenging. As a compromise, SNPMStat uses a small number of SNPs to impute untyped SNPs, which may omit additional correlations among SNPs. The factor that takes a dominant effect on the final performance is context dependent. Our results show that the method SNPMStat generally was not as good as ProbABEL, SNPTEST-BG, SNPTEST and BIMBAM, agreeing with the conclusion stated by Marchini and Howie [23], who demonstrated that more information was gained by use of all the available genotypes for imputation and of advanced population-genetics models than by modeling the difference between disease statuses. Plink uses multiple-marker tagging to impute untyped SNP but does not model specific population-genetic background. Compared to Plink, SNPMStat may offer improved power. Beagle uses empirical estimates as parameters. It may fit the model well under high LD level, but may mis-specify the model to some extent, particular when the sequence exhibits low LD level. Beagle was inferior to SNPMStat under medium LD level, which may be partially explained by that modeling the difference between disease statuses may gain more information than by use of biased population genetic model.

Results from real data sets further demonstrated that association with imputed genotypes could boost signals and improve power. The gene in which the signal was boosted is myelin basic protein (MBP) gene. This gene encodes a protein that is a major constituent of the myelin sheath of oligodendrocytes and schwann cells in the nervous system. MBP-related transcripts are also present in the immune system, which has long been recognized to influence drug addiction behavior [28], [29]. Molecular and cellular mechanisms of the nervous system react to addictive drugs to initiate and maintain patterns of drug-seeking behavior [30]. Given that MBP gene plays such an important role in nervous and immune system, it seems reasonable to speculate that MBP gene may influence smoking addiction.

In our simulation, the causal site was assumed to be known in reference set, which may not be the case in real applications. Nonetheless, our further analyses showed that the conclusions still hold even when the causal site was removed from reference sample. Additionally, the analysis carried out in current study focused on a SNP marker density of one SNP per 6kb. As denser genotyping chips have been developed, such as Affymetrix SNP 6.0 further studies considering more parameter settings appear to be warranted to better evaluate this issue.

## Materials and Methods

### Genotype simulation

The simulation of genotype data was similar to that adopted in our previous study [22]. Briefly, haplotypes covering a 250kb chromosomal region were simulated with uniformly distributed recombination rates across the region. The software Cosi [31] was used which implements a coalescent model to simulate haplotype. From the pool of simulated haplotypes, a diploid individual was generated by combining two randomly selected haplotypes. SNPs with MAF less than 0.05 were excluded from further analyses. Two-hundred and fifty approximately equally spaced SNPs, corresponding to a density of one SNP per kb were selected as the base SNP set on which all subsequent analyses were based. Two samples were generated, one reference sample and one test sample. In the reference sample, a total of 90 individuals were simulated, and genotypes at all the 250 SNPs were known. In the test sample, a total of 1,000 individuals were simulated, and genotypes at only a proportion of the 250 SNPs were known. We determined the marker density to be approximately one SNP per 6 kb (corresponding to 41 SNPs), and they were approximately equally spaced. The remaining SNPs were referred to as untyped SNPs and their genotypes were subject to be imputed by imputation methods.

A variety of parameter values were used to cover various biological conditions. Three recombination rates (between neighboring sites per generation): 1.0e-7, 1.0e-8 and 1.0e-9 were used to represent low, medium and high LD levels, respectively, consistent with a previous study [32]. In addition, effects of MAF were studied by binning untyped SNPs into one of five equally spaced intervals between 0.0 and 0.5 (0.05, 0.15, 0.25, 0.35 and 0.45).

### Phenotype simulation

We selected one SNP in reference sample but not in test sample as the causal site. Both quantitative and qualitative traits were simulated. For quantitative trait, the individual phenotype value was simulated according to the following equation
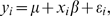
where 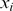 is the genotype value for the *i*th individual in an additive manner (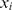 = 0, 1 or 2 for genotype 11, 12 or 22). 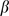 is the regression coefficient rendering the effect of causal SNP, and 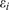 is a normally distributed residual effect. We assumed that the causal SNP explained 1.0% of the total phenotypic variation.

For qualitative traits, the individual phenotype was simulated by the following logistic regression equation
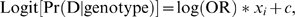
where OR is the odds ratio for the heterozygous genotype at the causal SNP, *c* is a constant rendering the specific case: control ratio. We set OR = 2 in this study.

### Real datasets

As an application, we analyzed a real genome-wide association study for smoking [33]. Basically, the data contain 840 unrelated Caucasian subjects (378 smokers and 462 non-smokers) each being genotyped by Affymetrix 500K array (Affymetrix, Santa Clara, CA). Detailed description of the data can be found in the reference [33]. We performed a genome-wide association scan with imputation. By monitoring the results, we then focused our attention on a 1.5 Mb genomic region at chromosome 18, named 18q23. We aimed to test association between the trait smoking and the genotyped or imputed genotypes in this region. Reference sample contained 60 unrelated CEU subjects from the HapMap Project (HapMap rel #21).

### Imputation-based Association Methods

Seven popular imputation-based association methods were investigated in this study: MACH2qtl/dat, SNPTEST, ProbABEL, Beagle, Plink, BIMBAM and SNPMStat. To control imputation quality, the respective quality-control (QC) cut-off recommended by each method was used. In cases where no cut-off was available, an empirical setting from extensive real applications would be adopted. The methods were briefly described below.

#### MACH2qtl/dat

MACH2qtl uses the dosages/posterior probabilities inferred from MACH as predictors in a linear regression framework to test the association with a quantitative trait. The command for association was: *mach2qtl –d sample.dat –p sample.txt –i sample.mlinfo –dosefile sample.mldose –probfile sample.mlprob>out.txt*. Instead, MACH2dat uses dosage in a logistic regression model to test association for a qualitative trait [21], [34]. The command was: *mach2dat –d sample.dat –p sample.txt –i sample.mlinfo –dosefile sample.mldose>out.txt*. The QC measure produced by *MACH2qtl/dat* is termed as rsq, which measures the squared correlation between imputed and true genotypes. We excluded SNPs with rsq less than 0.3 according to the authors' recommendation.

#### ProbABEL

Like MACH2qtl/dat, ProbABEL uses the posterior probabilities inferred from MACH as input to test the association [20]. It takes the uncertainty into consideration by including posterior dosage/probability to the design matrix of regression analysis (linear analysis for quantitative trait and logistic regression for qualitative trait). The command was: *palinear/palogist –pheno phenol.txt –info sample.mlinfo –dose sample.mldose*. For QC purpose, we again excluded SNPs with rsq less than 0.3.

#### SNPTEST

SNPTEST v 1.1.5 takes the results from the software IMPUTE as input, and uses “best-guess” genotype or posterior probabilities to test association. We included both tests into analysis to compare their relative performance. The command for qualitative trait was: *snptest –cases cases.gen cases.sample –controls controls.gen controls.sample –o out.txt –frequentist 1 –proper* and that for quantitative trait was: *snptest -controls controls.ped pheno.txt -o out.txt -qt -frequentist 1 –proper*. For analysis with “best-guess” genotype, we used the option“–*call_thresh* 0.9” to specify the best-guess genotype as the imputed genotype with posterior probability higher than 0.9. The resulting test was referred to as SNPTEST-BG. For analysis with posterior probabilities, it uses the distribution of the missing data conditional upon both the observed data and the values of the model parameters to correct likelihood-based procedure. As the QC measure, SNPTEST produces a “proper-info” to measure the relative statistical information about the parameters of interest. For QC purpose, we set a cut-off 0.4 to the measure, as used in extensive applications [35], [36].

#### Beagle

Beagle version 3.0 uses a hidden Markov model (HMM) to infer haplotype phase with both typed and untyped SNPs, and perform association test with the inferred haplotypes [18]. The command for inferring haplotypes was: *java –jar beagle.jar unphased = geno unphased = reference.txt markers = pos.txt missing = x nsample = s log = output*, and that for testing association was: *java –Xmx800m –jar beagle.jar data = output.bgl trait = pheno test = a*. Its current implementation analyzes qualitative traits only. As for QC measure, Beagle produces a measure r^2^ to estimate the squared correlation between the allele dosage with highest posterior probability and the true allele dosage for the marker. As the definition of r^2^ is similar to that of rsq in MACH, we again set the cut-off to 0.3.

#### Plink

Plink v 1.0.7 selects a set of proxy SNPs (using the reference sample information) and then phases these SNPs in both reference and test samples jointly. The association at a single imputed SNP is then examined by grouping haplotypes by flanking SNPs. The command was: *plink –file ref –merge test.ped test.map –pheno pheno.txt –mpheno i –proxy-assoc all –out out.txt*. The current implementation analyzes qualitative traits only. Plink produces a measure “info” which refer to how well plink manages, if at all, to impute the SNP. We set a cut-off 0.8 to this measure in accordance with the software's recommendation.

#### BIMBAM

BIMBAM v 0.95 is a Bayesian imputation-based association test [19]. It takes the imputation results from the software fastPHASE as input, and then uses Bayesian regression to test the association between imputed genotypes and phenotypes. The command to perform association was: *bimbam_lin test.geno –p test.pheno –g ref.geno –p 0 –pos pos.txt –o out.txt –i 1 –pval 1000*. Considering the heavy computation burden it would take, we performed 1,000 replicates of permutation to estimate an empirical*p*-value. We used the observed/expected dosage variance as its QC measure, which can be calculated by the following equation
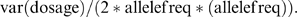
We set the QC cut-off as 0.3 according to a previous study [36].

#### SNPMStat

SNPMStat v 3.0 simultaneously fits a model of association and imputes genotypes by integrating inference of missing genotypes and estimation of odds ratio into a single likelihood framework. It derives the observed-data likelihood that properly reflects the biased nature of the case-control sampling and that incorporates appropriate external data. The maximization of the observed data likelihood leads to valid and efficient analyses of genetic effects. The command we used was: *SNPMStat –ur –no_remove –out out.txt*. SNPMStat produces a QC measure M_D (multilocus disequilibrium) to measure the correlation between the imputed SNP and a set of typed SNPs with the best prediction. We set the cut-off as 0.5 [37].

### Tests of association

We evaluated the performances of various association tests with three strategies: 1) test only the 41 genotyped SNPs; 2) test both the genotyped and imputed SNPs; 3) test the ideal genotypes (all the 250 genotyped SNPs).

For each parameter setting, 10,000 replicates were simulated to estimate power and type-I error rates. The powers at the single causal site and over the entire region were estimated. For the latter one, we took the minimal single site *p*-value over the region and adjusted it with Bonferroni correction to form the final *p*-value. Power was defined as the proportions of significant replicates at the nominal level 0.05. Under each MAF interval, we randomly selected 10 SNPs and took each of them in turn as causal SNP to simulate phenotype, and the averaged power was reported. We note that the power are comparable among methods only when their type-I error rates are comparable as well. Thus, for those conservative or liberal methods, we also calculated accuracy [38] and positive prediction value (PPV) [39], respectively. Accuracy of a test is defined as
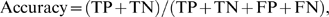
where TP (true positive) and FN (false negative) were the positive and negative results obtained when the causal site contributed to the phenotype. Similarly, FP (false positive) and TN (true negative) were the positive and negative results obtained when the causal site didn't contribute to the phenotype. Analogously, PPV is defined as